# Lignin-Derived Biomaterials for Drug Release and Tissue Engineering

**DOI:** 10.3390/molecules23081885

**Published:** 2018-07-27

**Authors:** Markus Witzler, Abla Alzagameem, Michel Bergs, Basma El Khaldi-Hansen, Stephanie E. Klein, Dorothee Hielscher, Birgit Kamm, Judith Kreyenschmidt, Edda Tobiasch, Margit Schulze

**Affiliations:** 1 Department of Natural Sciences, Bonn-Rhein-Sieg University of Applied Sciences, von-Liebig-Str. 20, D-53359 Rheinbach, Germany; markus.witzler@h-brs.de (M.W.); abla.alzagameem@h-brs.de (A.A.); michel.bergs@h-brs.de (M.B.); basma.elkhaldi-hansen@h-brs.de (B.E.K.-H.); stephanie.klein@h-brs.de (S.E.K.); dorothee.hielscher@h-brs.de (D.H.); edda.tobiasch@h-brs.de (E.T.); 2 Faculty of Environment and Natural Sciences, Brandenburg University of Technology BTU Cottbus-Senftenberg, Platz der Deutschen Einheit 1, D-03046 Cottbus, Germany; kamm@btu-cottbus-senftenberg.de; 3 Rheinische Friedrich-Wilhelms-University Bonn, INRES, Klein-Altendorf 2, D-53359 Rheinbach, Germany; 4 Kompetenzzentrum Holz GmbH, Altenberger Strasse 69, A-4040 Linz, Austria; b.kamm@kplus-wood.at; 5 Rheinische Friedrich Wilhelms-University Bonn, Katzenburgweg 7-9, D-53115 Bonn, Germany; j.kreyenschmidt@uni-bonn.de

**Keywords:** biomaterial, bone regeneration, drug release, hydrogel, lignin, multivariate data processing, osteogenesis, scaffolds, stem cells, tissue engineering

## Abstract

Renewable resources are gaining increasing interest as a source for environmentally benign biomaterials, such as drug encapsulation/release compounds, and scaffolds for tissue engineering in regenerative medicine. Being the second largest naturally abundant polymer, the interest in lignin valorization for biomedical utilization is rapidly growing. Depending on its resource and isolation procedure, lignin shows specific antioxidant and antimicrobial activity. Today, efforts in research and industry are directed toward lignin utilization as a renewable macromolecular building block for the preparation of polymeric drug encapsulation and scaffold materials. Within the last five years, remarkable progress has been made in isolation, functionalization and modification of lignin and lignin-derived compounds. However, the literature so far mainly focuses lignin-derived fuels, lubricants and resins. The purpose of this review is to summarize the current state of the art and to highlight the most important results in the field of lignin-based materials for potential use in biomedicine (reported in 2014–2018). Special focus is placed on lignin-derived nanomaterials for drug encapsulation and release as well as lignin hybrid materials used as scaffolds for guided bone regeneration in stem cell-based therapies.

## 1. Introduction

Materials used in biomedicine, such as polymers for drug encapsulation and tissue engineering scaffolds, are preferably produced from natural compounds, such as collagen-based composites for bone repair or alginates for controlled drug delivery. So far, numerous biopolymers have been studied in detail regarding their suitability for release materials and/or scaffold applications. Most of these materials are designed using polysaccharides, lipids and proteins [1,2,3].

**Figure 1:**
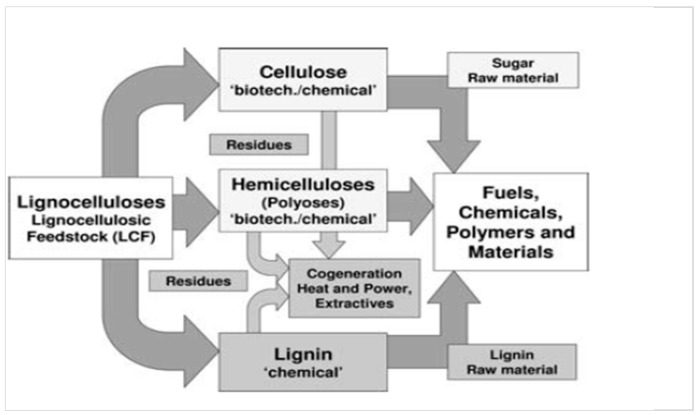
Lignocellulosic feedstock biorefinery [4]. Copyright 2018 WILEY-VCH Verlag GmbH & Co. KGaA, Weinheim.

Due to the development of biorefinery concepts for biomass treatment, starting about ten years ago, lignins have gained increasing interest in academic and industrial research. In particular, lignocellulose-rich feedstocks (LCF) are described for energetic and material exploitation as outlined in Figure 1 [4].

In the last few years, several pilot plants have been established and lignins are even commercially available to a limited extent [5,6,7]. Remarkable progress was made in lignin research, in particular isolation, structure analysis, functionalization and modification. Market analysis studies were published in 2017 and 2018, stating that the global lignin market is predicted to have an annual growth rate of about 2% until 2023 and an increasing total market size from US$ 904.04 Mio in 2017 to US$ 1021.57 Mio in 2023 [8,9]. According to the number of published patents and scientific studies, industrial applications so far are mainly directed toward lignin-based additives in concrete, dispersants, binders and resins. In contrast, studies including lignins for biomedical applications (release materials and/or scaffolds) are still very rare (Table 1 and Table 2).

In general, lignin could be used in many fields due to its dispersing, binding, complexing, and emulsion-stabilizing properties. However, lignin valorization is still challenging due to its complex and irregular chemical structure. Thus, the upgrading of lignin-derived materials toward applications in biomedicine is still limited to very few examples. The reproducible quality of the isolated structures required much effort. Today, sequential depolymerization via oxidative or reductive methods is one of the favored approaches to generate well-defined lignin fragments. In 2018, Sells et al. comprehensively reviewed the status quo of lignin depolymerization and upgrading approaches [10]. Among the few recently published studies on lignins in medicine are those of Vinardell and Santos: Vinardell et al. focused the bioactivity including antiviral and antimicrobial activity of lignins and their derivatives with special focus on their beneficial effects on human health [11]. Santos et al. reviewed recent developments to design and fabricate lignin-based nanostructures for biomedical applications [12].

The purpose of this review is to distinguish the lignin structural and morphological characteristics to exploit in stem cell-based approaches in regenerative medicine. A special focus is placed on biomaterials (scaffolds and drug release materials) used for mesenchymal stem cell (MSC) differentiation toward cardiovascular or bone tissue. Although a broad variety of scaffolds and release materials were studied in vitro and in vivo regarding their capacity to support tissue regeneration, there are just a very few studies including lignin-derived biomaterials so far. In contrast, numerous other natural biopolymers are studied in detail as well as synthetic polymers, glasses, ceramics, hydroxyapatite-based composites and nanostructured hybrids fabricated via conventional and additive manufacturing techniques [13,14,15].

## 2. Lignin Availability and Structure

### 2.1. Lignin Availability

**Figure 2:**
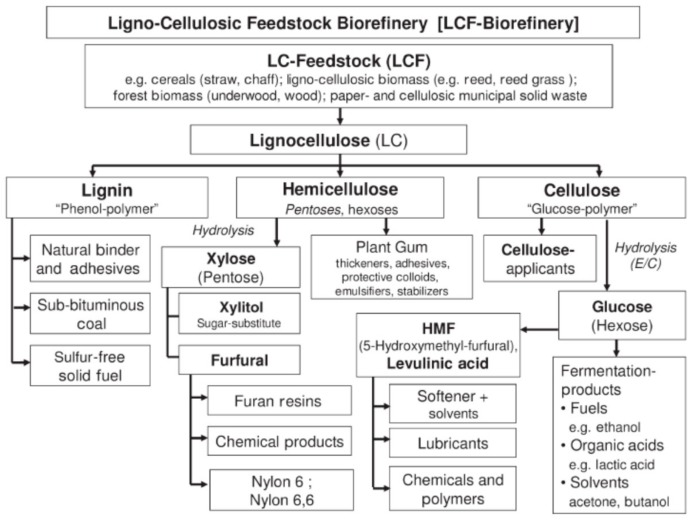
Products of a lignocellulosic feedstock biorefinery [7]. Copyright 2018 WILEY-VCH Verlag GmbH & Co. KGaA, Weinheim.

The assessment of biomass availability and quality is an important first step toward utilizing biomass for the development of value-added chemicals. Countrywide assessments of biomass resources have been performed for many single countries, i.e., U.S., Jordan, Malaysia, Turkey, China, India, Bangladesh [16,17,18]. However, there are no systematic studies regarding world-wide availability of lignocellulose-rich biomasses so far. One of the most important challenges is the handling of multiple biomass feedstock streams. A key step in processing lignocellulosic biomass is the separation of sugars from the lignocellulose. Several pre-treatments are applied for this: physical (grinding, milling), chemical (using acidic or basic aqueous media or ionic liquids), physicochemical (steam, hot water or ammonia fiber expansion) and biological fragmentation (via enzymes, fungi). Currently, the development of LCF biorefineries corresponds to the raw material available and focuses on a complete separation of the cellulose, hemicellulose, and lignin fractions using combinations of mechanical, chemical, and biotechnical methods (Figure 2).

Isolation methods such as Organosolv, acid, or alkaline steam-pressure processes are well-known methods which are commercially applied as the Organocell^®^, Alcell^®^, or Soda^®^ method [7]. Studies comparing different pulping and isolation techniques could show that the Organosolv process might be favorable to obtain lignins of good solubility and narrow molecular weight distribution [19,20,21].

Today, several pilot and demonstration plants have been developed using lignocellulosic feedstock (e.g., woodchips, straw) to produce bio-based building blocks (i.e., ethanol, acetic acid) and green coal in Germany (Leuna), The Netherlands (Bioprocess Pilot Facility), Austria (bioCRACK), Canada (GreenField), U.S. (Enchi Corp. Lebanon, NH, USA) and Australia (Microbiogen, Lane Cove West NSW 2066, Australia) [22,23]. Additionally, the first commercial biorefineries to produce ethanol from LCF, were recently established (e.g., DuPont in Nevada, Liberty™ Technology in U.S., POET-DSM in Iowa, Iogen Corp. in Canada, Raízen/Iogen in Brazil, Cellulac in Ireland) [24,25]. Currently, the pulp and paper industry produces the largest quantities of lignin (ca. 55 × 10^6^ tons per year). The most important industrial paper technology is the Kraft pulping process, leading to sulfur-containing degraded lignin fractions which are predominantly used as a secondary energy source. According to the market study, industrial producers of lignin and lignin-derived products around the world include the following: Domtar Corporation (Montreal, QC, Canada) southern pine-based BioChoice^®^), LignoTech Florida LLC (Fernandina Beach, FL, USA) southern yellow pine-based lignin utilizing a coproduct of RYAM’s sulfite pulping process, Borregaard’s technology (Sarpsborg, Norway), Weyerhaeuser Company (Seattle, WA, USA), collaboration with Lignol Energy Corp. (Vancouver, Canada), Stora Enso (LignoboostTM, Kraft lignin LineoTM^®^), GreenValue SA (sulfur-free lignin isolated form wheat straw, aqueous alkaline extraction), West Fraser, Domsj Fabriker (world’s 2nd largest producer of powder lignin), Changzhou Shanfeng Chemical Industry Co. Ltd. (Changzhou, Jiangsu, China), lignin polyether polyols), Nippon Paper Ind. Co. Ltd., (Tokyo, Japan) and The Dallas Group of America (Whitehouse, NJ, USA) Lignosulfonates [9].

### 2.2. Lignin Structure

**Figure 3:**
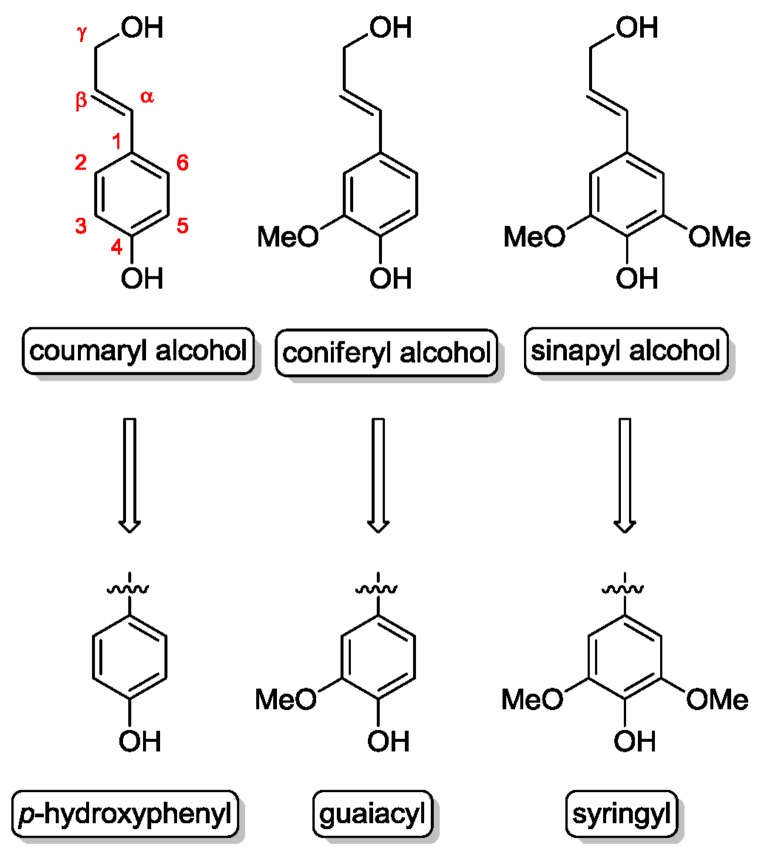
Structure of the three monolignol precursors and their corresponding fragments in the macromolecules.

**Figure 4:**
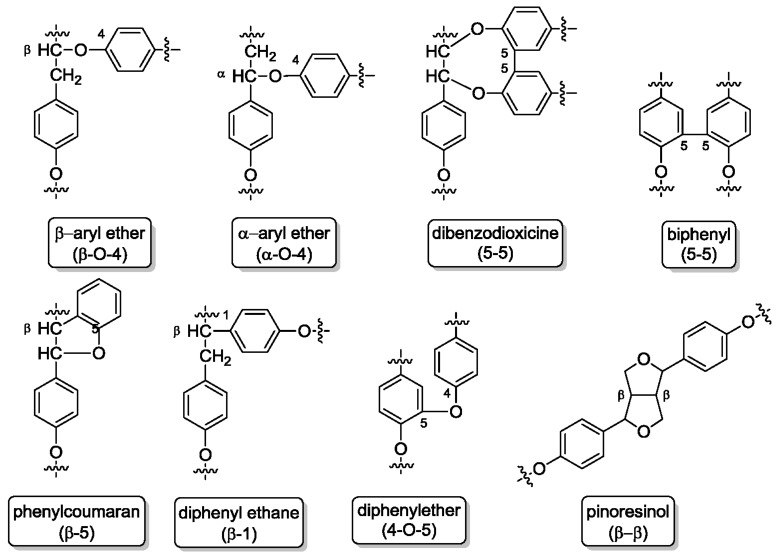
Lignin linkages: ether bonds, carbon-carbon bonds, and further linkages.

Lignin is a complex and irregular biopolymer containing randomly crosslinked phenylpropanoid units (cumaryl, coniferyl, and sinapyl alcohol) and found in plant secondary cell walls. Based on these monolignol units, the lignin building blocks *p*-hydroxyphenyl (H), guaiacyl (G) and syringyl (S) are formed (Figure 3). These building blocks are connected via several types of linkages (Figure 4), mainly ether bonds such as aryl- or phenyl ether and carbon-carbon bonds such as biphenyls, diphenyl ethane or pinoresinol.

In 2018, Gou et al. reported a detailed study on lignin biosynthesis systematically discussing the monolignol linkage formation [26]. Thus, during biosynthesis three endoplasmic reticulum-resident cytochrome P450 monooxygenases (C4H, C3′H, F5H) are required to generate the three different monolignol precursors. These three monooxygenases are tightly aligned on the cell membranes. However, they obviously do not directly interact with each other, but instead with two other membrane proteins, and thereby specifically control the connectivity of the phenylpropanoid–monolignols. In addition to the different biosynthesis pathways interfering with the formation of monolignol linkages, the pulping and isolation process and corresponding conditions (i.e., temperature, pressure, solvent, pH) significantly influence and change the lignin structure [19].

**Figure 5:**
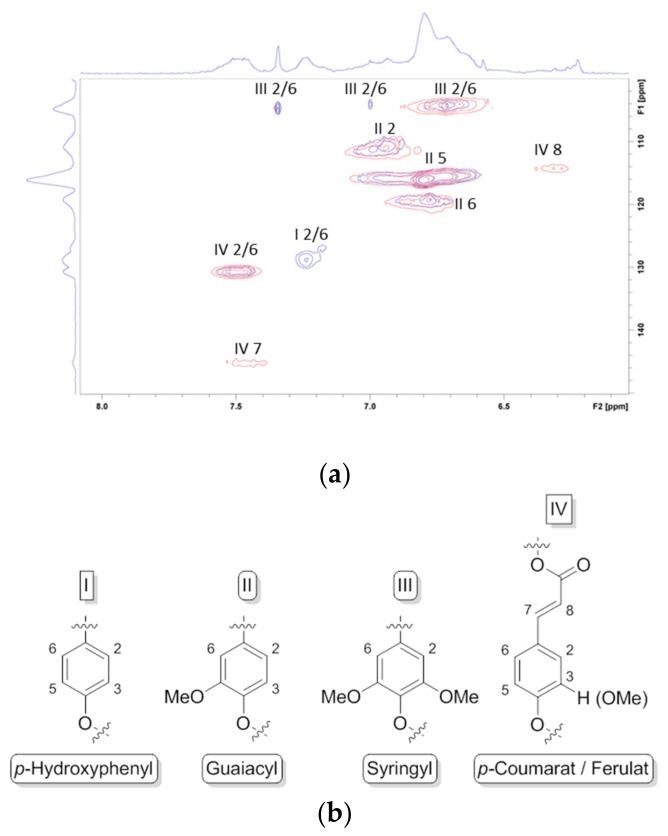
(**a**) HSQC NMR spectrum, aromatic region (dC/dH 100–150/6.0–8.0) of lignin samples obtained via Organosolv process from *Miscanthus X giganteus*. Comparison of leaf lignin (blue) and stem lignin (red) and (**b**) corresponding assigned lignin fragments. Copyright Springer 2018.

Numerous protocols have been developed to elucidate structural properties and compositional patterns that affect the processing of lignocellulose. In 2015, Lupoi and colleagues comprehensively reviewed lignin structure analysis studies, evaluating advantages and disadvantages as well as limitations of a broad number of analytical methods (i.e., infrared (FTIR), ultraviolet/visible (UV-Vis), Raman, and nuclear magnetic resonance (NMR) spectroscopy), mass spectrometry, chromatographic methods including size exclusion chromatography (SEC), gel permeation chromatography (GPC), high pressure liquid chromatography (HPLC), transmission and scanning electron microscopy, thermal analysis via differential scanning calorimetry (DSC) or thermographic analysis (TGA), as well as X-ray, neutron, and light scattering techniques [27]. The influence of various fractionation techniques (i.e., catalytic oxidative and reductive methods) on lignin structure and morphology is currently focused in numerous studies [28,29]. Thus, HSQC spectra of aromatic (dC/dH 100–135/5.5–8.5) and aliphatic (dC/dH 50–90/2.5–6.0) regions of lignin sample were discussed in detail by Chen and Vasilyev [30,31]. Analogue to these studies, we used the HSQC NMR spectroscopy to study differences in lignin structure obtained from various *Miscanthus X giganteus* genotypes. In addition, we also compared lignins isolated from stem versus leaves (Figure 5), showing a higher amount of *p*-hydroxyphenyl blocks in leaves, whereas stems have considerately more *p*-coumarat/ferulat blocks [21].

Cheng and co-worker reported a study combining small angle neutron scattering (SANS) and nuclear magnetic resonance analyses that enabled the description of detailed lignin structure in solution at a molecular level. They performed lignin solubilization studies (in DMSO-*d*_6_ and diluted aqueous NaOD) to investigate correlations between functional groups and its resulting ability to form aggregates via intermolecular interactions. Three lignins were investigated: two Kraft lignins (poplar wood, corncob) and one soda lignin. Intermolecular hydrogen bonding, non-covalent π–π interactions between phenyl rings, lignin chain conformation and the degree of branching were discussed considering operating forces for lignin solubilization [32].

The authenticity of natural products and biomass-derived polymers, such as lignin, concerns several different characteristics: quantitative and qualitative composition, geographical origin, type of raw material, producer, etc. Today, analytical platforms are developed to holistically prove the authenticity of natural products isolated from animal and/or plant raw materials. Thus, modern analytical methods are currently combined with multivariate data processing. Chemometric modeling of 2D NMR spectra (i.e., DOSY, HSQC, HMBC) are reported using principal component analysis (PCA), independent component analysis (ICA), multivariate regression (PLS), and various discriminant analysis methods (i.e., LDA, FDA, PLS-DA). Quantitative characteristics (molecular weight, content of active ingredients and impurities, pharmacological activity, etc.) and qualitative properties (plant origin, genotype, phenotype, manufacturer) can be determined based on spectrometric and chromatographic profiles, as we could recently show for heparins of different origin using 2D NMR and SEC data [33]. This approach is universal and can be applied to other methods and products as well. Empirical techniques have evolved into statistical approaches (i.e., FTIR, NMR). Sluiter and Krasznai comprehensively reviewed the studies published so far regarding compositional analysis of lignocellulosic biomass and corresponding isolated compounds including lignin [34,35]. Besides FTIR, Raman, and NMR spectroscopy, neutron and X-ray scattering are appropriate methods to deliver the required data quantity and quality to be used for compositional analysis of biomass-derived compounds including lignin [36]. Various ball-milled *Miscanthus X giganteus* phenotypes were analyzed by Haffner et al. using near-infrared spectroscopy. PLS regression analysis was used to predict plant extract components such as glucan, xylan, arabinan, acetyl, Klason lignin, total ash, and ash after extraction. Milling to uniform sizes is required since particle size significantly influences the reproducibility of the data [37]. In another study, Hayes et al. reported compositional analysis using NIR and UV-Vis spectroscopy of Miscanthus. In particular, Miscanthus particle size and moisture content were varied using different pre-treatment methods (wet-chopping, air-drying, grounding, sieving). Determined data include glucose, xylose, and Klason lignin [38]. The same spectroscopic methods (NIR, UV-Vis) were applied by Everard et al. to estimate the gross calorific value of ground Miscanthus and two coppice willow stem samples [39]. Sugarcane lignocellulose was analyzed using diffuse reflectance near-infrared spectroscopy and multivariate calibration by Chong et al. to determine ash, lignin, and carbohydrate composition data [40].

## 3. Lignin Antioxidant Capacity and Bioactivity

### 3.1. Lignin Antioxidant Capacity

Due to their polyphenolic structure, lignins possess antioxidant activity. Kraft lignin from wood sources in pulp industry was reported to be as efficient as vitamin E to protect the oxidation of corn oil [41]. Most antioxidant effects of lignins are considered as derived from the scavenging action of their phenolic structures on oxygen containing reactive free radicals. Although there are several options to study antioxidant activities of naturally occurring phenolic compounds, the 2,2-diphenyl-1-picryl-hydrazyl-hydrate (DPPH) method using 1,1-diphenyl- 2-picrylhydrazyl as a reactive free radical, is recognized as appropriate for lignin structures, analogue to radical scavenging ability of flavonoid and catechin structures. The reactivity of DPPH is far lower than that of oxygen containing free radicals (OH, RO, ROO and O2), and unlike them the interaction rate is not diffusion-controlled. Dizhbite et al. compared DPPH and ABTS (2,2′-azino-bis(3-ethyl benzothiazoline-6-sulphonic acid) methods and found rather good conformity [42]. As their free radical scavenging ability is facilitated by their hydroxyl groups, the total phenolic concentration could be used as a basis for rapid screening of antioxidant activity [43]. The total phenolic levels can be determined based on their chemical reducing capacity relative to gallic acid or by using the Folin–Ciocalteu reagent [44,45]. Son and Lewis observed DPPH inhibition effects for methylated lignin derivatives [46]. Barapatre and colleagues studied in detail activity differences of aliphatic and free phenolic hydroxyl groups confirming that the radical scavenging activity of phenolic compounds depends on the hydrogen abstraction rate [47]. In our studies we could confirm the proposed mechanism and improve the antioxidant activity of Kraft lignin extracts up to 68% compared to 55% for literature values. In addition, the Kraft lignins were compared to Organosolv lignins obtained from beech wood and grasses [21].

### 3.2. Lignin Antimicrobial Activity

The literature describing the microbial properties of lignins has grown rapidly in the last decade, comprehensively reviewed by Espinoza-Acosta et al. [48]. In addition to their effects on antioxidant activity, phenolic hydroxyl and methoxy groups have been reported to be biologically active. Thus, numerous investigations have suggested that lignins can be applied to stabilize food and feedstuffs due to their antioxidant, antifungal, and antiparasitic properties [49]. Commodity products with antioxidant or antimicrobial properties, such as sunscreen lotions, biocomposites, and clothes that use lignin as a natural ingredient have been prepared, and their characterization has shown promising results [11]. Dumitriu and Popa confirmed in their studies that the main determining factor of the antimicrobial effect of lignin correlates with phenolic fragments and the nature of further functional groups as well as specific side chain constitution. Typically, the presence of a double bond in α, β positions of the side chain and a methyl group in the γ position grants the phenolic fragments the most potency against microorganisms [50].

Primary antimicrobial study performed with Kraft lignin extracts showed that purification strongly influenced the lignin bioactivity against *S. aureus* and *L. monocytogenes* (gram-positive bacteria) and *E. coli* (gram-negative bacteria) [21]. Lignin nanoparticles incorporated in polylactic acid (PLA) revealed an innovative capacity to inhibit the bacterial growth along the time [49]. The decrease of oxidative and inflammatory damage to the kidney in streptozotocin-induced diabetic rats due to lignin-derived lignophenols was reported by Sato et al. [51]. Similar to these results, low molecular weight lignins were tested regarding their potential as anti-emphysema agents in vitro [52].

Additionally, other properties such as anticarcinogenic, apoptosis-inducing antibiotic, and anti-HIV activities have been reported for lignin-carbohydrate complexes (LCCs). The toxicity of free radicals contributes to cellular damage such as DNA and protein damage, inflammation processes, tissue injury and cellular apoptosis which could cause cancer development. Barapatre et al. showed an antioxidant and antidiabetic efficiency of modified alkali lignin. The antidiabetic property has been investigated in terms of in vivo glucose movement inhibition and α-amylase inhibition. The modified samples effected the α-amylase inhibition and an increased glucose binding efficiency was evaluated by decreased glucose diffusion [53]. Studies of Hasegawa et al. were focused on the evaluation of lignosulfonic acids in relation to α-glucosidase activity. Their results suggest a suppression of blood glucose via inhibition of the α-glucosidase and intestinal glucose absorption. Here, lignosulfonic acid is a reversible and non-competitive inhibitor [54]. Besides antioxidant and antidiabetic properties lignins seem to influence the secretion of apolipoprotein B and cholesterol levels and thus play a role in obesity control. This was investigated by Norikura presenting lignophenols to a human hepatocellular carcinoma cell line leading to reduced levels of apo-B and cholesterol [55].

Furthermore, lignins are also studied regarding their antiviral capacity. Gordts et al. investigated lignosulfonic acid, regarding HIV antiviral activity, and revealed lignosulfonic acid to be a potent inhibitor of the HIV replication. They also prevent an uptake of the virus by CD4T cells from persistently infected T cells in vitro [56]. The antiviral activity of lignin products is also shown for human α-herpes viruses including herpes simplex viruses (CMV and herpes simplex virus (HSV-1 and HSV-2). Ligno-carbohydrates seem to inhibit the viral binding, penetration and replication [57,58]. The inhibition of the replication of herpes simplex virus (HSV) was studied by Andrei and colleagues [59]. They found, that topical tenofovir, a microbicide effective against HIV, inhibits herpes simplex virus-2 replication.

Henry and colleagues reported that lignins do also show anticoagulant effects. In particular, they investigated low molecular weight lignins regarding their inhibiting influence on thrombin and factor Xa through allosteric disruption of the enzymatic apparatus [60]. In accordance to these results, Mehta et al. published similar studies focusing sulfated β-O4 lignins, which act as an allosteric inhibitor of thrombin to reduce fibrinogen cleavage resulting in a reduction of platelet activation [61].

## 4. Lignin-Derived Biomaterials for Drug Encapsulation/Release and Tissue Engineering

### 4.1. Gels and Hydrogels for Drug Encapsulation and Release

**Figure 6:**
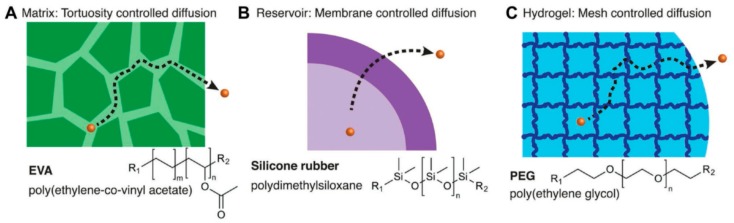
Examples of controlled release platforms. (**A**) Matrix tortuosity-controlled diffusion; (**B**) Membrane-controlled diffusion; (**C**) Hydrogels [62]. Copyright 2018 John Wiley and Sons.

Considerable interdisciplinary research efforts have been focused on the design of biomaterials for drug delivery applications. However, kinetically controlled release remains a challenge due to several open questions regarding the chemical and biological criteria that limits drug delivery. Within the last fifty years, a broad variety of encapsulation and release materials have been designed to release bioactive drugs for an extended period and (in best case) initiate specific interaction with the host to control the released drug amount. Basic release mechanisms include: matrix tortuosity-controlled diffusion, membrane-controlled diffusion for small molecules and hydrogels via mesh size and network swelling (Figure 6) [62].

**Figure 7:**
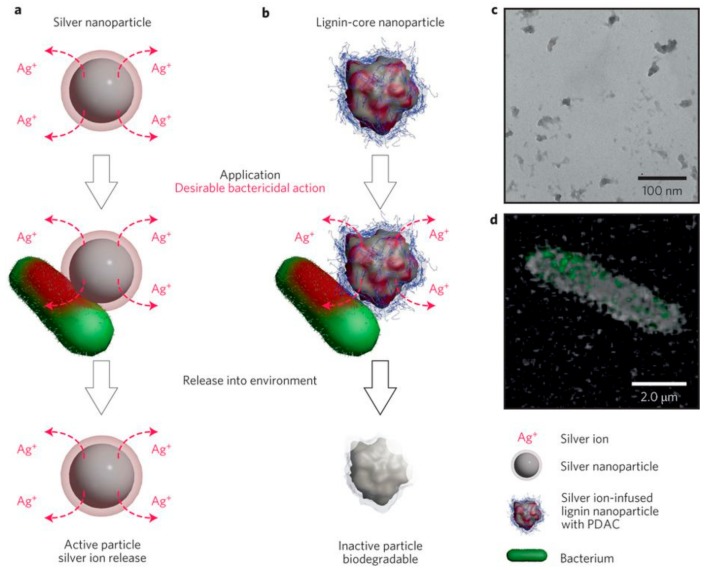
An environmentally benign antimicrobial nanoparticle based on a silver-infused lignin core. (**a**) General mechanism for the antimicrobial action of common AgNPs via the release of Ag^+^ ions, which continues post-utilization; (**b**) Mechanism of antimicrobial action of Ag^+^ ion-infused EbNPs with a cationic polyelectrolyte coating that facilitates electrostatic attraction between the EbNPs and negatively charged cell walls. In contrast to AgNPs, EbNPs are depleted of Ag^+^ ions during their application, minimizing their post-utilization activity; (**c**) TEM micrograph of as-synthesized EbNPs in the size range of 40–70 nm; (**d**) Confocal microscopy image of EbNPs with polyelectrolyte coating adhering to the cell membrane of *E. coli.* [63]. Copyright 2018 Springer Nature.

In the past decade, the number of research groups working on “lignin valorization” steadily increased, including lignin-based gels and hydrogels for controlled and/or sustained release of pharmaceutical drugs and compounds such as pesticides used in agriculture. In 2015, Velev and colleagues first reported the development of antimicrobial nanoparticles with biodegradable cores, prepared from Indulin AT® lignin, loaded with silver cations and coated with a cationic polyelectrolyte (polydiallyldimethylammonium chloride, PDAC) (Figure 7). The lignin-derived nanoparticles showed biocidal activity on both gram-negative and gram-positive human pathogens as well as quaternary amine-resistant bacteria at significantly lower silver concentrations compared to conventional reagents such as silver nanoparticles or silver nitrate while leaving an inactive biodegradable particle after the release, thus having a smaller impact on the environment [63].

In June 2018, Österberg and colleagues reported another “breakthrough in lignin research”: the synthesis of colloidal cationic lignin nanoparticles to encapsulate enzymatic biocatalysts to be used for esterification in aqueous media. The catalyst is immobilized (“spatially confined”) within the lignin-derived colloidal particles [64]. Recent approaches in the development of lignin-derived nanoparticles generated for biomedical applications were reviewed by Beisl and Shi [65,66]. In particular, nanostructured lignin hydrogels, their synthesis, characterization and possible applications were reported by Thakur and Kai [67,68]. Table 3 summarizes lignin-derived encapsulation materials (macro- and nanosized derivatives) studied regarding their bioactivity and release performance.

Thus, lignin is shown to be a promising resource for biodegradable, bioactive materials, either for agricultural formulation of e.g., pesticides or fertilizers studied by Chowdury or in colloidal form as “green” alternative for metallic nanoparticles in pharmaceutical drug delivery [69,70]. Lignin colloidal spheres are also tested as sunscreen additives in cosmetics. Qian et al. produced sun screen lotions with lignin spheres of about 50 nm that reached a sun protection factor (SPF) of about 15 for UVA radiation. However, the SPF seems to be dependent on both size and extraction method of the lignin spheres [71]. Figueiredo et al. developed lignin nanoparticles via self-assembly during dialysis. They incorporated iron or iron oxide into the lignin particles to get magnetic particles and performed drug release studies of the poorly water-soluble drugs Sorafenib^®^ and Benzazulene^®^ on pure lignin particles. All variations of particles showed low cytotoxicity. Drug release kinetics of hydrophobic drugs depends on the pH of the release medium, because of accelerated lignin particle degradation in more alkaline media: at both pH 5.5 and 7.4 (aqueous buffer with 10% fetal bovine serum) more than 90% of the drug was released in the first 6 h. However, in pure water, the drug could be retained in the particles for 15 days [72]. 

Nanospheres of enzymatic hydrolysis lignin were prepared by Xiong et al. via self-assembly by adding a non-solvent to the lignin solution. The group was able to prepare spheres in the range of 190–590 nm with a good stability over 30 days of storage. However, albeit proposing a possible carrier function, no experiments regarding drug release of biocompatibility have been performed in this study [73]. In 2017, Dai et al. reported the synthesis of lignin nanoparticles as a green carrier for the sustained Resveratrol^®^ drug release. The group encapsulated the hydrophobic drug Resveratrol^®^ together with Fe_3_O_4_ nanoparticles for possible use in targeted cancer therapy. The particles showed a sustained drug release of 80% over 4 days. Both, in vitro and in vivo biocompatibility and anticancer tests of particles with and without magnetic particles revealed no adverse effects on cells or mice [74]. Just recently, Li et al. prepared polyelectrolytic microparticles from quaternary ammonium lignin and sodium dodecyl benzenesulfonate via particle precipitation. They loaded the particles with hydrophobic insecticide Avermectine during the precipitation step and investigated both drug release and UV protection capability of the microparticles. The release into 1:1 methanol:water was somewhat sustained with about 80% of the drug being released in 72 h. The anti-photolysis properties of the lignin proved to be very good, after 96 h of UV irradiation 85% of the drug was still preserved in the spheres [75]. The same group also reported lignin droplets in a Pickering emulsion coated with polyuria for a sustained Avermectine release. Here, the drug was loaded to the droplet during emulsion before the coating step. Polyurea-coated lignin spheres proved to sustain the Avermectine release into 4:1 ethanol:water less than pure polyurea coatings (85% in 72 h and 50% in 72 h, respectively). The authors found that the lignin-polyurea layer is much more porous than the pure polyuria layer due to the 3D structure of the lignin. However, UV protection of the microparticles proved to be very good, after 120 h of irradiation, more than 75% of the drug was preserved [76].

In addition to nanospheres, Wang et al. prepared a hybrid hydrogel of montmorillonite and a lignin-derived graft copolymer (lignin-*g*-acrylamide-isopropyl acrylamide) that could be used as an effective agent for the removal of dyes or other chemicals from aqueous solutions. At room temperature and neutral pH, the hybrid hydrogel outperformed other hydrogel systems in the adsorption of methylene blue by a factor of 4–10 [77]. Further examples for lignin-based composites are cellulose-lignin hybrid hydrogels, which were prepared by Ciolacu et al. in 2012. Here, cellulose and lignin were crosslinked with epichlorohydrin resulting in porous materials after lyophilization. Drug release from those gels was investigated using different polyphenols as model drugs. Loading was performed by swelling the dried gels in polyphenol solution. Both swelling and subsequent drug release is dependent on lignin content in the gel. Higher lignin ratios lead to both a higher swelling and a faster and higher release of polyphenols. However, drug release has only been monitored for about 10 h, resulting in a maximum of 30% of the released drug [78].

Besides spheres and gels, lignin-based films were synthesized and tested for biomedical applications. Kosikova et al. reported thin films of a lignin-polypropylene blend to have improved antioxidant properties against thermos-oxidative degradation. Furthermore, the use of lignin as stabilizer in plastics as positive effects on the protection of mice DNA against oxidation damage due to lignin’s scavenging effects [79]. Gregorova et al. also prepared thin films, using polyethylene and lignin nanoparticles. The lignin acts as antibacterial agent and its effectiveness against *E. coli* and *S. aureus* was found to be equal to other bactericides such as bronopol or chlorohexidine. Moreover, the addition of the lignin did not alter the mechanical properties of the films [80].

### 4.2. Lignin-Based Scaffolds for Tissue Engineering

Research on biomaterials for tissue engineering and regenerative medicine covers various interdisciplinary aspects: depending on the final application, scaffolds must fulfill several very different, sometimes even contradictory requirements. Bone regeneration scaffolds are required to show sufficient mechanical stability when implanted, combined with controlled degradability, and replaced by natural bone thereby avoiding toxic degradation products. So far, bone replacement materials include: autologous transplants (source: chin area, retro-molar region, iliac crest, trabecular bone), allogeneic transplants (availability via bone banking), xenogeneic transplants (temperature or chemical pre-treatment), alloplastic (hydroxyapatite, tri-calcium phosphate, ceramics, polymers based on α-hydroxyl acid). Novel stem cell-based approaches allow individualized patient-specific solutions. Biomaterials are specified by: biocompatibility according to ISO standards including long-term studies, stability against physiological media (pH, temperature), mechanical stability depending on specific application (e.g., stress/strain, elongation, impact moduli etc.), corrosion resistance (for metallic components), residual-free metabolization in case of biodegradable materials, and appropriate technical functionality according to specific application. First step in cell-scaffold interaction are cell adhesion processes related to intensive interaction on cell–biomaterial surface and interfaces. These interactions strongly depend on surface polarity (hydrophilic versus hydrophobic surfaces), surface roughness and topography. The scaffold development starts with polymer synthesis using state-of-the-art polymerization techniques to achieve well-defined porous structures to enable cell ingrowth. Polymer bulk and surface must be tailored to meet needs of the natural environment. Scaffold surface polarity and topography must be adapted to the cell shape to support cell adhesion, proliferation and growth [81,82].

**Figure 8:**
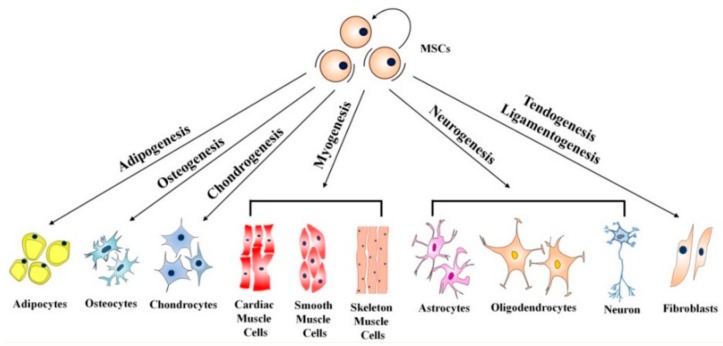
The differentiation potential of mesenchymal stem cells [13]. Copyright 2018 Springer eBook.

In the following, the focus is placed onto stem cell-based approaches in bone regeneration using scaffold materials that in detail influence the differentiation and proliferation of MSC. They are found in all adult mesenchymal tissues and play a role in the maintenance of tissue homeostasis and repair by allowing renewal of the cellular stock. MSCs can be isolated from both human and animal sources. Adipose tissue is a rich and promising source of these cells. Adipose-derived stem cells (ASCs) are often effective and safe, and have been used in preclinical and clinical studies for both autologous and allogeneic transplantation. The potential use of stem cell-based therapies for repair and regeneration of various tissues and organs provides an important alternative therapeutic solution for the treatment of many diseases [83,84,85,86]. MSCs have the potential to differentiate into multiple mesenchymal derived lineages (Figure 8).

Adipogenesis leads to adipocytes and osteogenesis to osteocytes. Similar, chondrogenesis will end with chondrocytes. Myogenesis will generate cardiac, smooth, and skeletal muscle cells and neurogenesis will lead to astrocytes, oligodendrocytes, and neurons. After tendogenesis and ligamentogenesis fibroblasts are produced. MSCs can be isolated from different body parts for instance; with amniotic fluid, umbilical cord, dental tissue, bone marrow, peripheral blood, skin, and adipose tissue being the most common sources. Following the isolation process, the MSCs can be differentiated toward osteoblasts using a suitable differentiated medium. Appropriate scaffolds seeded with osteoblasts are considered to become the best choice for future bone regeneration.

**Figure 9:**
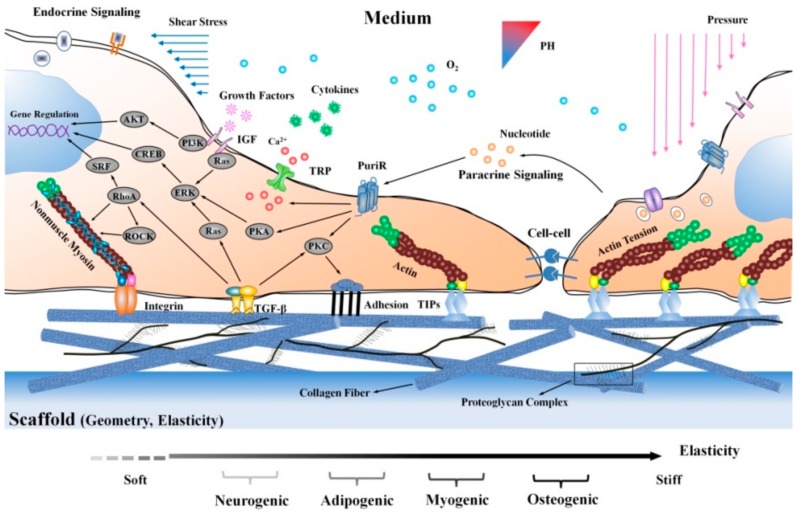
Stem cell and their natural microenvironment. Factors influencing the stem cell niche can be roughly categorized in three groups: physical and mechanical factors such as shear forces, elasticity and topography, cellular issues such as immune and nerve cells, nearby blood vessels and neighboring stem cells and soluble factors such as oxygen, glucose, hormones, growth factors or signaling molecules [13]. Copyright 2018 Springer Verlag.

Stem cells are affected by their microenvironment which is defined by extracellular matrix properties such as elasticity and geometry. Molecules with similarities to the extracellular matrix, for instance transforming growth factor-β (TGF-β), tension induced proteins (TIPs), integrins and transient receptor potential (TRP), can regulate cytoskeleton tension successively followed by gene expression and focal adhesion though the activation of a series of mechanical transduction events. Also, various soluble factors such as extracellular nucleotide, growth factors and cytokines influence stem cell fate. Mechanical forces such as shear stress and blood pressure influence stem cell proliferation and differentiation from the media side of the niche as well as chemical and physical factors such as pH or oxygen (Figure 9).

Delella and her colleagues reviewed the state-of-the-art regarding the control of mesenchymal stem cell manipulation process prior to their use. In particular, they studied the effect of the endocrine disruptor bisphenol A (BPA) on MSC fate and tried to explain the mechanisms by which BPA interferes with adipogenesis and increases adipose tissue in humans [87]. Since lignin-derived compounds are also discussed to become a BPA substitute, their potential effect on MSC fate must be investigated more in detail [88].

**Figure 10:**
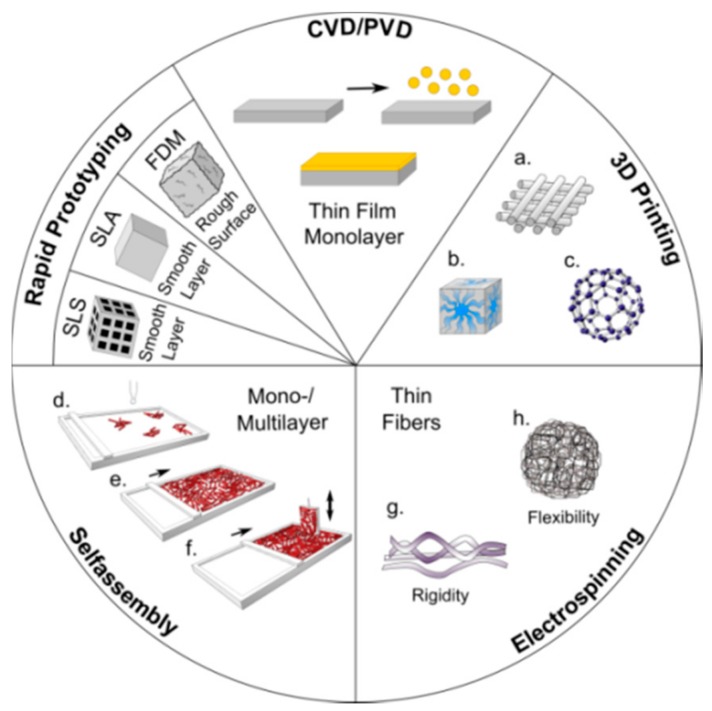
Fabrication methods used for the development of nanostructured scaffolds for tissue engineering applications: 3D printing, electrospinning, rapid prototyping and self-assembly techniques such as Langmuir-Blodgett.

So far, a large variety of materials are studied regarding their ability to support MSC differentiation, proliferation and growth, both in vitro and in vivo: natural polymers (i.e., gelatin, alginate, silk, and collagen), synthetic polymers, glasses and ceramics or HA-based composites and hybrids prepared via conventional techniques or as nanostructured materials via additive manufacturing [89]. Nanofabrication techniques for scaffold generation include rapid prototyping (RP) methods such as selective laser sintering (SLS), selective laser ablation (SLA), fused deposition modeling (FDM), chemical and physical vapor deposition (CVD, PVD), 3D printing methods resulting in tailor-made layered, cubic and spherical structures (Figure 10).

In addition, self-assembly methods such as Langmuir-Blodgett technique for monolayer formation and electrospinning are used for scaffold fabrication of bone and vascular tissue [15]. As already discussed for the hydrogel production, lignin-derived nanoparticles (spheres, rods, films) are prepared for various applications, such as encapsulation of drugs and dyes or nanofiber production [64,65,66,67]. Xu and colleagues studied and reviewed the potential of various lignins for 3D printing in very detail including various processing parameters [90].

Among the large number of materials reported so far for stem-cell-based tissue engineering, only very few developed from lignocellulose feedstock and/or lignin derivatives. First, Rekola et al. in 2009 reported the osteoconductivity of heat-treated wood bone implants. Without investigating lignin, the group found that a heat treatment of wood increased the biological behavior of such implants, with a higher temperature resulting in improved in vitro osteoconductivity [91]. An alginate-lignin composite aerogel has been prepared by Quraishi et al. The group mixed solutions of alginate and lignin and used CO_2_ induced gelation and foaming to produce aerogels with µm-sized interconnecting pores. Aerogels showed low stiffness in the range of fibrous tissue, but no cytotoxic effects on mouse fibroblast-like L929 cells in vitro [92]. Farhat et al. produced a variety of different polysaccharide-based composites using a reactive extrusion process. Starch, hemicellulose or lignin were crosslinked with citric acid, and the corresponding hydrogels were characterized by means of swelling, mechanical strength and degradability. Swelling is depending on pH but also on the amount of citric acid used as cross-linker. Degradation rates were studied at physiological condition for 15 days. Degradation could be reduced using additional catalysts during polymer extrusion. Dynamic mechanical analysis revealed that the hydrogel degradation induces significant reduction in the compressive modulus [93,94]. Agarose-lignin hydrogels were prepared and studied regarding their mechanical behavior by Techato and colleagues. Agarose solubilized in water forms a gel with a rigid network, resulting on a three-dimensional porous structure. Furthermore, agarose hydrogels may be polymerized in situ thereby allowing the hydrogel to acquire the required shape. Lignin obtained from oil palm empty fruit bunches is used to generate lignin-agarose hydrogel with epichlorohydrin as the cross-linking agent. The gel strength of composite lignin-agarose hydrogel was studied by texture analysis [95]. Very recently, Morganti discussed the potential of chitin and lignin to be used as natural scaffold materials imitating the extracellular matrix (ECM). The authors prepared composites consisting of nanoscaled lignin and chitin nano-fibrils of high surface area-to-weight ratios [96]. Wang et al. reported reinforced chitosan microfibers prepared by adding various amounts of lignin during the spinning process. They showed that addition of 3–5% lignin improves tensile strength and stiffness of chitosan. The authors predict good biocompatibility without proving this by experimental data [97]. In 2015, Anwer et al. prepared various poly (lactic acid) (PLA)—lignin composites and studied their mechanical properties. They found that a lignin filler content of up to 15% negatively influences the tensile strength of the composites, and that the crystallization of PLA is also slowed down [98]. Just recently, Spiridon and Tanase also published a study on PLA-lignin composites. Here, an addition of up to 7% lignin microparticles led to a decrease in tensile strength; however, adding 7–15% lignin increased the tensile strength. Additionally, biocompatibility of the composites was tested on SaOS-2 cells with no observed adverse effects [99]. Lignin-based copolymers comprised of lignin-poly(ε-caprolactone-*co*-lactide) were synthesized via solvent-free ring-opening polymerization and subsequently spun into blend nanofibers. The copolymers were blended with either polycaprolactone (PCL) or poly (l-lactic acid) (PLLA) during electrospinning. The spun mats were evaluated regarding their mechanical properties, antioxidant activity and biocompatibility. The PCL-blends were mechanically improved; however, the stability of the PLLA-blends was slightly decreased. Antioxidant activity and biocompatibility on the other hand were improved. PLLA-blends showed an increased viability and proliferation of NIH/3T3 fibroblasts, making them interesting candidates for tissue engineering applications [100]. Erakovic et al. prepared a bioactive coating for titanium implants. The coatings comprised of hydroxyapatite (HA) and Organosolv lignin in various ratios were deposited onto the implants electrophoretically and were sintered afterwards. The coatings showed good biocompatibility, and, when doped with silver during deposition, even a good antibacterial effect against *S. aureus* [101]. Table 4 summarizes examples of lignin-derived materials reported to be candidates for scaffold utilization.

There are also several studies on polyurethanes (PU) used in tissue engineering. Due to the broad variety of available (stiff or flexible) polyol and isocyanate components for polyurethane synthesis, their internal structure and morphology can be tuned to resemble natural bone and promote tissue ingrowth [102,103]. Biodegradable water-based shape memory polyurethane scaffolds for bone regeneration were prepared by Wang and colleagues using 3D printing (via low-temperature fuse deposition). Supramagnetic iron oxide nanoparticles were incorporated to promote osteogenic induction. Scaffolds seeded with hBMSCs showed improved osteogenesis compared to conventional PU scaffolds [104]. Since lignin-based polyurethanes are already available in various compositions with tunable mechanical stability and degradability, lignin-PU in future will become favored candidates to be studied in detail regarding their ability to be used for both, biodegradable tissue engineering scaffolds and drug encapsulation materials [105,106].

**Figure 11:**
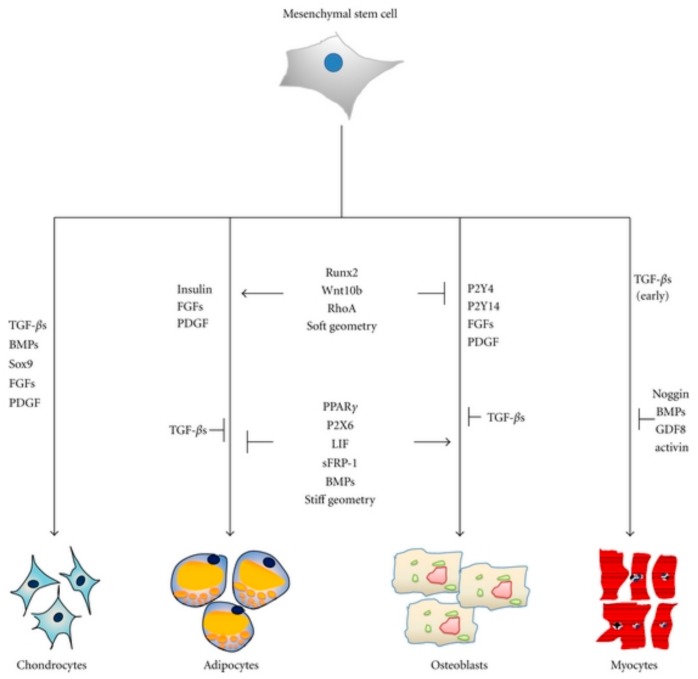
Key molecules regulating adipogenesis and osteogenesis in mesenchymal stem cells. Runx2, Wnt10b, RhoA, and soft geometry can induce osteogenesis while inhibiting adipogenesis [108]. Copyright 2018 under the Creative Commons Attribute License.

Within the last five years, calcium phosphate cements and hydroxyapatite-derived hybrid materials gained increasing interest since they are shown to combine scaffold function with additional ability for sustained release, in detail studied for several different drugs including low molecular weight (i.e., antibiotics, anticancer drugs) and high molecular weight compounds (i.e., proteins, growth factors) and ions (i.e., Ca, Sr, Si, Zn, Mg) [107]. Due to their osteoconductivity and injectability they are already used as bone grafts. Moreover, their low-temperature setting reaction and intrinsic porosity allow drug incorporation and release. Osteogenesis of MSCs is known to be influenced by a broad number of osteoinductive and osteoconductive compounds, respectively, such as growth factors. Recently, we could show that stem cell differentiation toward bone is guided by various purinergic receptors (P2X and P2Y), Figure 11 [108,109,110].

Thus, controlled release of corresponding P2 ligands (agonists, antagonists) encapsulated and kinetically controlled released during MSC differentiation could be used to tailor MSC differentiation into cardiovascular and bone tissue, respectively [110]. First hybrid materials based on HA and polysaccharide (agarose derivatives) have been prepared to be used as appropriate scaffold for MSC differentiation and guided ligand release [111]. Here, lignin could be used in the future to partially substitute the polysaccharide components and thereby improve mechanical stability as shown for the agarose/lignin composites. 

## 5. Conclusions and Perspectives

Highly advanced biomaterials are required for stem cell-based approaches in tissue engineering. Initial studies on lignin-derived composites do confirm its potential to be used for scaffolding and/or drug release development. Antioxidant and antimicrobial capacity of lignin extracts allows a broad variety of potential applications, such as drug in cancer therapy. Although the isolation of well-defined lignin fractions is still challenging, the intrinsic bioactivity will be the driving force for successful implementation of lignin-derived biomaterials in medicine. Regarding lignin structure analysis, compositional data processing will become more important to specify detailed structural differences due to biomass source, plant genotypes or isolation conditions. Future efforts in scaffold development for bone regeneration will likely be directed toward nanostructured hydroxyapatite-derived hybrid materials imitating the complex natural bone composition. While conventional manufacturing methods are mainly based on chemical induction of differentiation via growth factors and cytokines, concentrating on altering material properties such as substrate stiffness and topography to mimic the stimuli stem cells receive in their natural niche is becoming more of a focus in the manufacturing process. Once the first promising lignin-derived nanocomposites are reported, they will be studied in detail regarding their ability for kinetically controlled release to guide stem cell differentiation and finally improve tissue regeneration.

## Figures and Tables

**Table 1:** Number of scientific publications on “lignins” refined by “lignin in drug release” and “lignin scaffolds” according to Web of Science, searched on 20 June 2018.

Publication Years	“Lignin”	“Lignin and Drug Release”	“Lignin and Scaffolds”
2014	2856	3	23
2015	3269	5	25
2016	3672	10	35
2017	3893	12	39
2018	1783	8	13

**Table 2:** Number of patents specifying “lignin”, refined by “lignin in drug release” and “lignin scaffolds”, respectively, according to World Intellectual Property Organization (WIPO), searched on 20 June 2018.

Filing Year	“Lignin”	“Lignin and Drug Release”	“Lignin and Scaffolds”
2014	5877	474	683
2015	5766	440	601
2016	5912	449	601
2017	5264	412	488
2018	1691	153	183

**Table 3:** Lignin-derived systems for biomedical applications: drug release and antibacterial use.

Application	Matrix Type	Encapsulation Method and Active Ingredient	Results	References
drug release	lignin nanoparticles from Indulin AT	nanoparticle flash precipitation with subsequent silver ion infusion and polyelectrolyte coating	>95% release of silver ions in 24 h and antibacterial effect against *E. coli*, *P. aeruginosa* and *Rastonia* sp.	Richter et al. 2015 [63]
drug release	lignin nanoparticles from LignoBoost^TM^ softwood Kraft lignin	incorporation of poorly water-soluble Sorafenib^®^ and Benzazulene^®^ during particle formation via polarity change	poorly water-soluble drugs are released upon degradation of the particles; the water-soluble drug could not be incorporated into the nanoparticle; low cytotoxic effects on cancer cell lines: MDA-MB-231, MCF-7, PC3-MM2, Caco-2 and non-tumor cells: KG1 and EA.hy926 endothelial cells	Figueiredo et al. 2017 [72]
drug release	lignin nanospheres from enzymatic hydrolysis lignin	no drug loading	lignin nanoparticles with tunable size can be produced via self-assembly	Xiong et al. 2017 [73]
drug release	lignin nanoparticles from alkaline lignin	incorporation of Resveratrol^®^ during particle formation via polarity change	about 80% drug released into phosphate buffer saline (PBS) after 4 days	Dai et al. 2017 [74]
drug release	polyelectrolyte microparticles of quaternary ammonium lignin-sodium dodecyl benzenesulfonate (lignin from pine alkali lignin)	loading of hydrophobic Avermectine during particle precipitation	release of ~80% Avermectine into methanol:water (1:1) after 72 h; good UV protection of the drug (85% preserved after 96 h UV irradiation 30 W, 310 nm)	Li et al. 2018 [75]
drug release	lignin droplets in W/O Pickering emulsion coated with polyurea	loading of hydrophobic Avermectine in emulsion before droplet coating reaction	release of 85% of Avermectine into 4:1 ethanol:water after 72 h; lignin-polyurea coatings were more porous than pure polyuria layers, which showed a more sustained release; UV protection of lignin coatings was good (>75% preserved after 120 h irradiation 30 W, 310 nm)	Pang et al. 2018 [76]
drug release	montmorillonite/lignin-acrylamide-isopropyl acrylamide copolymer	adsorption of methylene blue from aqueous solution	effective removal of dyes from aqueous solutions over multiple sorption/desorption cycles	Wang et al. 2017 [77]
drug release	crosslinked cellulose-lignin hydrogels (steam expansion lignin, aspen wood)	swelling of gel in polyphenol solution	a higher lignin content leads to a faster drug release, up to 30% in 10 h	Ciolacu et al. 2012 [78]
antibacterial effect	lignin nanoparticles in polyethylene films (Björkman lignin from beech wood flour)	none	lignin particles exhibit antibacterial effect against *E. coli* and *S. aureus* in the same order of magnitude as other antibacterial agents such as bronopol and chlorohexidine	Gregorova et al. 2011 [80]

**Table 4:** Lignin-derived scaffold for possible bone tissue engineering applications.

Aim	Matrix Type	Additional Ingredients	Results	References
osteoconductivity	heat-treated birch wood	none	heat treatment of wood increases osteoconductivity	Rekola et al. 2009 [91]
scaffold fabrication	alginate-lignin aerogel (lignin from wheat straw by enzymatic hydrolysis)	none	fluid uptake in Tris-HCl buffer of >1600%, good biocompatibility	Quraishi et al. 2015 [92]
scaffold fabrication	starch, lignin (from Kraft lignin) or hemicellulose	none	hydrogels produced by reactive extrusion show pH dependent swelling behavior (water uptake at pH 9: from 400 to 1400%); the amount of citric acid used as cross-linker also influences both swelling and degradation of the hydrogels. Additional catalysts used during extrusion slow down degradation	Farhat et al. 2017 [94]
scaffold fabrication	agarose-lignin composites (lignin from Kraft black liquor)	none	crosslinked agarose-lignin hydrogels exhibit enhanced mechanical properties compared to pure agarose gels	Techato et al. 2018 [95]
influencing mechanical properties	lignin-chitosan microfibers	none	improving mechanical properties of chitosan fibers by adding 3% lignin	Wang et al. 2016 [97]
influencing mechanical properties	poly(lactic acid) with lignin as filler (Kraft lignin)	none	lignin as filler does not decrease storage modulus, but inhibits PLA crystallization	Anwer et al. 2015 [98]
influencing mechanical properties	poly(lactic acid) with up to 15% lignin as filler (Organosolv lignin from birch wood and Kraft lignin from softwood)	none	higher lignin content leads to higher tensile strength, but also slightly decreased water sorption capacity. Organosolv lignin yields slightly better mechanical results; good biocompatibility against SaOS-2 cells regardless of lignin type	Tanase et al. 2018 [99]
influencing mechanical properties	lignin-based copolymer/polyester blend nanofibers (alkali lignin)	none	mechanical improvement dependent on polyester, good antioxidant activity and biocompatibility against NIH/3T3 fibroblasts	Kai et al. 2017 [100]
bioactive coating for implants	hydroxyapatite/lignin composite coatings on titanium (Organosolv lignin)	doping of silver for antimicrobial effect	HA coatings on Ti were non-cytotoxic to peripheral blood mononuclear cells; Ag-doped coatings showed antibacterial behavior against *S. aureus*	Erakovic et al. 2014 [101]
